# Dimethylfumarate inhibits microglial and astrocytic inflammation by suppressing the synthesis of nitric oxide, IL-1β, TNF-α and IL-6 in an in-vitro model of brain inflammation

**DOI:** 10.1186/1742-2094-7-30

**Published:** 2010-05-19

**Authors:** Henrik Wilms, Jobst Sievers, Uta Rickert, Martin Rostami-Yazdi, Ulrich Mrowietz, Ralph Lucius

**Affiliations:** 1 Department of Neurology, University of Heidelberg, Im Neuenheimer Feld 400, 69120 Heidelberg, Germany; 2 Department of Anatomy, Olshausenstr. 40, University of Kiel, D-24098 Kiel, Germany; 3 Psoriasis-Center, Department of Dermatology, Venereology, and Allergology University Medical Center Schleswig-Holstein, Campus Kiel, Schittenhelmstrasse 7, D-24105 Kiel, Germany

## Abstract

**Background:**

Brain inflammation plays a central role in multiple sclerosis (MS). Dimethylfumarate (DMF), the main ingredient of an oral formulation of fumaric acid esters with proven therapeutic efficacy in psoriasis, has recently been found to ameliorate the course of relapsing-remitting MS. Glial cells are the effector cells of neuroinflammation; however, little is known of the effect of DMF on microglia and astrocytes. The purpose of this study was to use an established in vitro model of brain inflammation to determine if DMF modulates the release of neurotoxic molecules from microglia and astrocytes, thus inhibiting glial inflammation.

**Methods:**

Primary microglial and astrocytic cell cultures were prepared from cerebral cortices of neonatal rats. The control cells were treated with LPS, an accepted inducer of pro-inflammatory properties in glial cells, and the experimental groups with LPS and DMF in different concentrations. After stimulation/incubation, the generation of nitric oxide (NO) in the cell culture supernatants was determined by measuring nitrite accumulation in the medium using Griess reagent. After 6 hours of treatment RT-PCR was used to determine transcription levels of iNOS, IL-1β, IL-6 and TNF-α mRNA in microglial and astrocytic cell cultures initially treated with DMF, followed after 30 min by LPS treatment. Moreover, we investigated possible involvement of the ERK and Nrf-2 transduction pathway in microglia using western blot analysis.

**Results:**

Pretreatment with DMF decreased synthesis of the proinflammatory mediators iNOS, TNF-α, IL-1β and IL-6 at the RNA level in activated microglia and astrocytes in vitro, associated with a decrease in ERK phosphorylation in microglia.

**Conclusions:**

Collectively, these results suggest that the neuroprotective effects of DMF may be in part functionally attributable to the compound's ability to inhibit expression of multiple neuroinflammatory mediators in brain of MS patients.

## Background

Despite growing knowledge regarding its pathogenesis, and approval of different classes of immunomodulating drugs, multiple sclerosis (MS), a chronic CNS-disease characterized by neuroinflammation and demyelination, remains one of the leading causes of disability in young adults [1,2]. Approved therapies for MS include interferon-β (IFN-β) and glatiramer acetate as first-line, and natalizumab and mitoxantrone as second-line [3] treatments. The first-line drugs reduce disease activity and progression only moderately and the second-line drugs are characterized by serious possible side effects such as development of progressive multifocal leukencephalopathy for natalizumab and cardiotoxicity for mitoxantrone. Moreover, all drugs mentioned above require either subcutaneous or intramuscular (first-line drugs) injections or intravenous infusions (second-line drugs). Thus, there is a high demand for orally active, highly efficious drugs with limited side effects. One such candidate might be dimethylfumarate (DMF), which is the active ingredient of an oral formulation of fumaric acid esters with proven effectiveness in patients with chronic plaque psoriasis, a dermatological disorder with immune dysfunction [4,5]. In experimental autoimmune encephalomyelitis (EAE), an animal model of MS, DMF and its hydrolysis product monomethylfumarate (MMF) inhibit the disease course, inhibit macrophage activation in the spinal cord and increase expression of interleukin-10 [6]. In patients with relapsing-remitting MS, DMF reduced new inflammatory lesions in serial MRI-scans in a Phase II trial [7]. These positive results have led to the initiation of two international multi-center double blind and placebo-controlled Phase III - studies to further test the efficacy of the drug: Patients are currently recruited for the DEFINE ("determination of the efficacy and safety of oral fumarate in relapsing-remitting MS") and the CONFIRM ("comparator and an oral fumarate in relapsing-remitting MS") studies.

Despite the demonstrated benefits, there are only few experimental data available concerning DMF-mediated influences on the glial - especially microglial - environment in the CNS. This could be of interest because part of the effect could be mediated via interference with pro-inflammatory microglia cell functions: In multiple sclerosis infiltration of T cells into the nervous system initiates a complex immunological cascade consisting of epitope spreading, which triggers new attacks, and activation of the innate immune system (e.g. microglia, dendritic cells) which leads to chronic inflammation. Studies using PET-scans have shown clusters of activated microglia in MS in vivo [8]. Autopsies studies have shown that diffuse axonal injury with profound microglia activation is found even in normal-appearing white matter [9]. Microglia are distributed throughout the CNS as a network of resting immunocompetent cells derived from the monocyte/macrophage lineage. The cells become rapidly activated in response to injury or in the presence of pathogens and - like other tissue macrophages in the first line of host defense - play a pivotal role as phagocytic, antigen-presenting cells. However, it is also this efficient defensive action that makes them potentially neurotoxic cells. By releasing various kinds of noxious factors such as proinflammatory cytokines (i.e. TNF-α, IL-1β, IL-6) or proinflammatory molecules like nitric oxide (NO) microglia may potentiate damage to CNS cells [10-12]. IL-1β [13] and NO [14] are expressed in chronic active plaques in MS patients. NO reacts with superoxide anion to generate peroxynitrite, a highly reactive molecule capable of oxidizing proteins, lipids and DNA; and which mediates microglial toxicity to oligodendrocytes [14] NO can trigger immune cascades that further enhance inflammatory-mediated CNS damage: Increased concentrations of NO can lead to enhanced expression of chemokine (C-C- motif) ligand 2 (CCL2) on astrocytes which, in turn, leads to infiltration of CD11b cells and additional tissue damage [15]. The cytokine TNF-α is an important factor in the regulation of neuronal apoptotic cell death. TNF-α mRNA expression in blood mononuclear cells is correlated with disease activity in relapsing-remitting MS [16,17], while high IL-6 levels in the CNS correlate with the development of EAE in rats [18]. IL-6 is detected in acute and chronic active MS plaques from the brain of patients [19]. Transgenic mice overexpressing IL-6 develop acute neurodegenerative pathology, including ataxia, tremor and seizures suggestive of a profound effect of this cytokine on many components of the CNS [20]. Astrocytes are also producers of a variety of cytokines including IL-1, IL-6 and TNF-α [21].

Given this, there is little doubt that activated glia cells can inflict significant damage on neighbouring cells in MS. The effects of DMF in EAE give rise to the hypothesis that the therapeutical benefit of DMF might be at least partly due to an influence on the glial, especially microglial environment. Our study therefore addresses the question if DMF might influence the release of the aforementioned inflammatory mediators from activated microglial cells and astrocytes in vitro. Since nitric oxide [22] and IL-1β [23] are induced at the protein level in macrophages and microglia through the activation of ERK pathway, we moreover investigated if DMF modulates this signalling pathway. Furthermore, the role of nuclear factor erythroid 2-related factor 2 (Nrf2), a transcription factor implicated in neuroprotection, was also determined.

## Methods

### Microglial cell culture

For all experiments Wistar rats were used, which were bred and kept under constant conditions (12 h/12 h light/dark cycle) in the animal house of the University of Kiel.

Microglial cells were prepared from rostral mesencephali and cerebral hemispheres of 2-day-old Wistar rats with slight modifications as described previously [24]. Meninges, hippocampi and choroid plexus were removed from the brains and cortices and mesencephali were minced, mechanically dissociated by trituration using fire-polished Pasteur pipettes, followed by enzymatic digestion with trypsin (from bovine pancreas, type III, Sigma) and DNAse I (Roche). Suspended mixed brain cells were plated in culture flask (75 cm^2^, Sarstedt) in 10 ml growth medium (Dulbecco's modified Eagle's Medium, DMEM; no. 41965, Invitrogen), supplemented with 10% (v/v) FBS (fetal bovine serum, Invitrogen), 1% (v/v) penicillin (10,000 U/ml)/streptomycin (10 mg/ml, PAA). All cells were cultured in a humidified atmosphere enriched with 5% CO_2_. Free floating microglial cells were collected from the medium of primary cell cultures from neonatal rat cerebral cortex after 10 days as detailed elsewhere [25].

Prior to replating microglial cells for the different experiments, cell number and viability was estimated by trypan-blue exclusion. Viable cells were seeded onto 96-well-microtiter plates (200,000 cells/well for NO measurement) or 12-well-culture plates (1,000.000 cells/well for realtime RT-PCR, 1,500.000 cells/well for Western blot) and grown for 24 hours at 37°C in a humidified atmosphere enriched with 5% CO_2_.

Astroglial cell cultures were prepared according to a slightly modified method of McCarthy and De Vellis [26] from brains of 2-day-old Wistar rats as described previously [27]. This method allowed the preparation of nearly pure cultures of astrocytes with a microglial cell portion, identified by immunocytochemical OX-42 staining, of <0.5%. In brief, cerebral hemispheres were freed from the meninges, the hippocampus, and the choroid plexus. The cortices were dissociated mechanically (using fire-polished Pasteur pipettes) and enzymatically (with trypsin (type III) and DNAse I), and suspended cells were plated into culture flasks (25 cm^2^) in growth medium (DMEM supplemented with 10% (v/v) FBS (56°C heat-inactivated), 1% (v/v) penicillin/streptomycin and 0.1% (v/v) amphotericin B. For the elimination of neurons, oligodendrocytes and fibroblasts on day 9 in vitro, the culture flasks were shaken by rotatory shaking (200 rpm, 24 h), and treatment with 1 *μ*M 5-fluoro-2'-deoxyuridine (Sigma, Deisenhofen, Germany) for 48 h. Further enrichment in subcultures was obtained by (i) eliminating contaminating microglial cells by a preplating procedure, and (ii) by seeding the cells at low cell density (5000 cells/cm^2^), which further reduced the number of contaminating microglial cells.

### Drug treatment

For the induction of microglial activation, lipopolysaccharide (LPS, 10 ng/ml, from Salmonella typhimurium, Sigma), a bacterial endotoxin and a generally accepted inducer of pro-inflammatory properties, was added to the control groups. The experimental groups were moreover incubated in the initial experiments with different concentrations (1 μM - 100 μM) of DMF (solved in dimethylsulfoxide, DMSO, Sigma), leading to a concentration of 10 μM DMF (= 0,1% DMSO) used in further experiments.

For stimulation experiments the experimental groups were moreover pre-incubated with 10 μM DMF (30 minutes) before LPS was added for a further 6 - 24 hours. The control groups remained untreated. This concentration was used since it most probably reflects an appropriate concentration with regards to the in vivo situation: In 2009 our group detected a level of 5.5 mg/l of the mercapturic acid of DMF after oral intake of 240 mg DMF in 24 h urine of psoriasis patients [28].

PD 98059 was dissolved in DMSO (Sigma) to yield a 10 mM stock solution and was diluted to a final concentration of 10 μM in the experiments.

### Measurement of nitrite production

The nitrite concentration in the culture supernatant was used as a measure of NO production. After stimulation/incubation, the generation of NO in the cell culture supernatants was determined by measuring nitrite accumulation in the medium using Griess reagent (1% sulfanilamide and 0,1% *N*-(1-naphthyl)-ethylenediamine dihydrochloride in 5% H_3_PO_4_, Sigma, Germany). One hundred microliters of culture supernatant and 100 μl Griess reagent were mixed and incubated for 5 minutes. The absorption was estimated in an automated plate reader (SLT reader 340 ATTC) at 540 nm. Sodium nitrite (NaNO_2_, Merck, Darmstadt) was used to generate a standard curve for quantification. Background nitrite was substracted from the experimental value. Results were obtained from three separate measurements of identically treated wells/drug, and the data are derived from four independent experiments.

### Quantitative RT-PCR

Microglia and astrocytes were washed three times with PBS (4°C). RNA was isolated with the TRIZOL reagent, digested by DNase (Promega, Mannheim, Germany) to destroy contaminating DNA, and cDNA was synthesized with RevertAid™ H Minus M-muLV Reverse Transcriptase (Fermentas, St. Leon-Rot, Germany). One microgram of total RNA was reverse transcribed into 50 ng/μl cDNA by random hexamer primer (no. 27216601, Amersham Biosciences). Ten nanograms of cDNA were used for PCR amplification. Quantitative reverse transcriptase PCR was performed in three replicates of each sample using TaqMan primer probes (Assays on demand; Applied Biosystems, Foster City, CA, USA) on an ABI Prism 7000 thermocycler using assays-on-demand and chemistries as recommended by the manufacturer (all Applied Biosystems). The PCR signal of the target transcript in the treatment groups was related to that of the control by relative quantification. The 2^-ΔΔCT ^method was used to analyze the relative changes in gene expression [29]. The housekeeping gene 18S rRNA was used as internal control to normalize the PCR for the amount of RNA added to the reverse transcription reactions and the target gene expression was normalized to the control. Data are expressed as percent change of gene expression relative to LPS-stimulated controls (= 100%).

TaqMan assays had the following identification numbers:

18s: Hs 99999901, iNOS: Rn 00561646, TNFα: Rn 99999017, IL-6: Rn 00561420, IL-1β: Rn 00580432.

### Western blotting

For western blot analysis, 750,000 - 1,500,000 microglial cells were treated with 10 ng/ml LPS in the presence or absence of 10 ng/ml DMF and after a pre-incubation (30 min.) with PD98059. The cells were washed twice with PBS, harvested in PBS by scraping, and centrifuged. Cellular protein was isolated from the cell pellet using 100 μl lysis buffer (128 mM Tris/HCl, 4.6% SDS, 10% glycerine, 0.005% bromphenolblue, 25 μl/ml mercaptoethanol, pH 6.8), boiled for 5 min (95°C), and insoluble material was removed by centrifugation at 10,000 × g at 4°C for 5 min. Protein aliquots (5 μg each) were resolved by 12.5% SDS-PAGE and western blotted with phospho-specific antibodies against ERK1/2 (Cell Signalling Technology, MA, USA) overnight at 4°C according to the manufacturer's protocol. For western blot analysis of Nrf2 translocation, the nuclear fraction of microglia cells was separated using an NE-PER kit purchased from Pierce, Rockford, IL (USA). Protein aliquots of lysate (20 μg for Nrf2 and reloaded with Lamin A/C as loading control) were detected using a primary antibody against Nrf2 (Santa Cruz). Antibody binding was detected via enhanced chemiluminescence (Amersham Pharmacia Biotech, Essex, UK).

### Statistical analysis

All experiments were performed at least three times and the results presented are from representative experiments. The significance of the difference between experimental and control groups was analyzed using analysis of variance (ANOVA) followed by the Bonferroni test using GraphPad Prism 3 Software. An α-level of 0,05 was used for statistical significance.

## Results

### Dimethylfumarate reduces mRNA levels of iNOS and NO-synthesis

**Figure 1:**
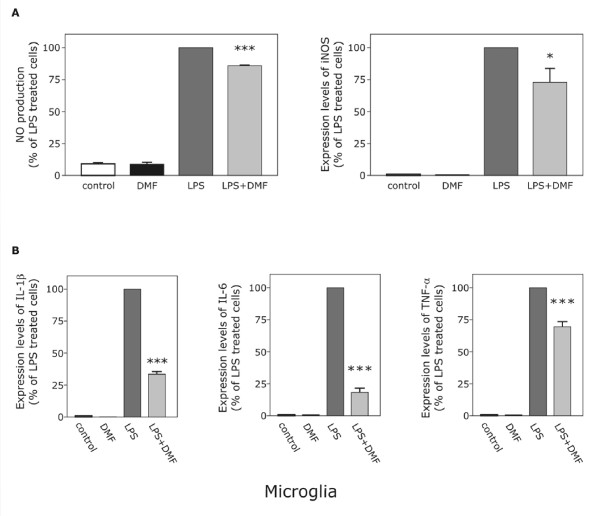
**Dimethyl fumarate inhibits LPS-induced mRNA expression of NO (A), iNOS, Il-1β, Il-6 and TNF-α (B), respectively, in cultivated microglia. **LPS was added to microglia cells 30 min after the addition of dimethyl fumarate (10 μM) to analyze its effect on mRNA expression after 6 hours of treatment. mRNA expression was analyzed using TaqMan real-time RT-PCR and results for cells treated with dimethyl fumarate were compared to those of cells stimulated solely by LPS. 18s RNA (a housekeeping gene) was used as an internal control. The data were assessed from 3 independent experiments, each run in triplicate. Asterisks (* = p < 0.05; *** = p < 0.001), indicate significant differences compared to cells stimulated solely by LPS (ANOVA followed by the Bonferroni test).

Pretreatment of cells with different nontoxic concentrations of DMF before LPS activation led to a significant and dose-dependent reduction of nitric oxide synthesis in microglia (Fig. 1A): After 24 hours there was a significant inhibition (p < 0.001) of LPS-induced increase in nitrite levels (100%) after cotreatment with dimethyl fumarate (85,8 ± 0,3%). The suppressive influence of DMF was also visible at the iNOS mRNA level (73% ± 10,8%) compared to the LPS control.

### Dimethylfumarate reduces mRNA levels of the pro-inflammatory cytokines IL-1β, IL-6 and TNF-α in microglia

To determine if DMF also suppresses the expression of IL-1β, IL-6 and TNF-α, real-time RT-PCR analysis was done. The transcription levels of all investigated cytokines were upregulated after LPS treatment (100%). This increase was significantly (p < 0.001) inhibited by treatment with dimethyl fumarate for IL-1β (33,4% ± 2,0%), TNF-α (69,5% ± 4,1%) as well as IL-6 (18,2% ± 3,4%) in microglia (Fig. 1B).

### Astrocytic synthesis of IL-1β, IL-6 and TNF-α is inhibited by dimethylfumarate

**Figure 2:**
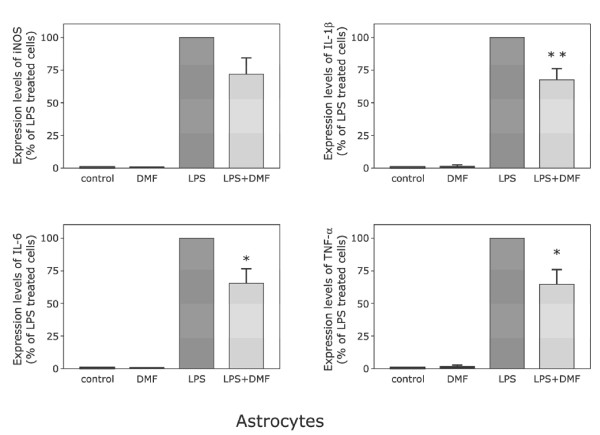
**Dimethyl fumarate inhibits LPS-induced mRNA expression of iNOS (A), Il-1β (B), Il-6 (C) and TNF-α (D) in astrocytes. **LPS was added to astrocytes 30 min after the addition of dimethylfumarate (10 μM), its effect on mRNA expression was analyzed after 6 hours of treatment. mRNA expression was analyzed using TaqMan real-time RT-PCR and results for cells treated with dimethyl fumarate were compared to those of cells stimulated solely by LPS. 18s RNA (a housekeeping gene) was used as an internal control. The data were assessed from 3 independent experiments, each run in triplicate. Asterisks (* = p < 0.05; ** = p < 0,01), indicate significant differences compared to cells stimulated solely by LPS (ANOVA followed by the Bonferroni test). Note that the reduction of iNOS mRNA (Fig. 3A) is not statistically significant.

As demonstrated by real-time PCR, DMF suppresses the expression of IL-1β, IL-6 and TNF-α mRNA levels in pure astrocytic cultures. As expected, transcription levels of all investigated cytokines were upregulated in astroglial monocultures used as controls after LPS treatment (100%). These increases in specific mRNA levels were significantly (p < 0.001) inhibited by treatment with DMF for IL-1β (67,5% ± 8,8%; Fig. 2), IL-6 (65,3% ± 11,2%) as well as TNF-α (64,5% ± 11,4%). iNOS mRNA was only moderately and not statistically significant reduced (71,9% ± 12,5%).

### Inhibition of the ERK-pathway is involved in the decreased synthesis of proinflammatory microglial molecules induced by dimethylfumarate

**Figure 3:**
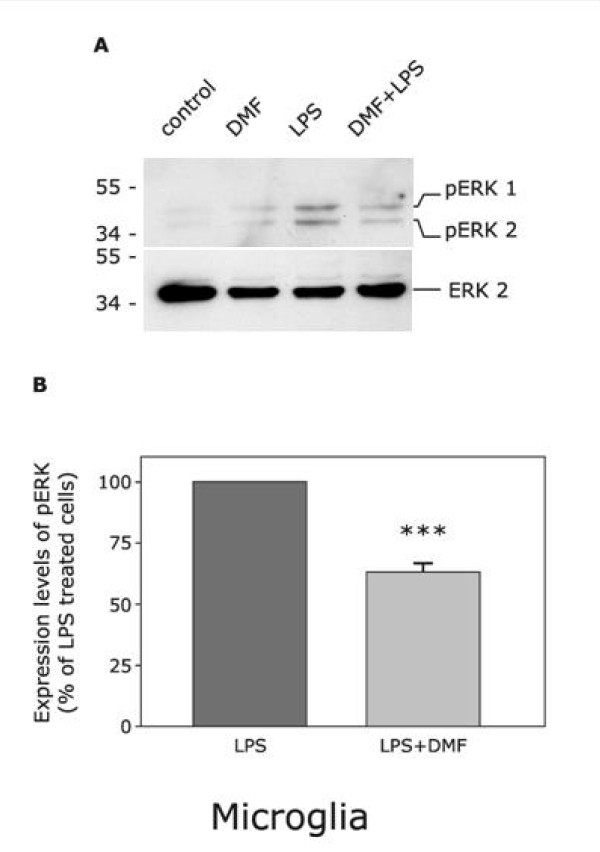
**DMF inhibition of ERK phosphorylation in LPS-activated microglia. **Results are compared to microglial cultures stimulated solely by LPS. Results are detected by western blot after 5 minutes of treatment. Activated ERK (phospho-ERK) species were detected by immunoblot analysis with antibodies specific for the phosphorylated forms of the kinase (A). The amount of protein loaded in each lane was confirmed by measurement of the amount of ERK using antibody against the unphosphorylated form of ERK. (B) Blots were subjected to densitometry and normalized to the signal of the respective *pan*-MAPK antibody. Results are representative of 3 independent experiments (*** = p < 0.001).

Considering our findings that DMF inhibited the synthesis of NO, IL-1β, IL-6 and TNF-α in microglial cell cultures, we investigated a possible involvement of the ERK transduction pathway: Western blot analysis confirmed that, in comparison to LPS-stimulated microglial cells used as controls, cotreatment with DMF inhibited phosphorylation of ERK by 37,2% (62,9 ± 4,0; Fig. 3).

### DMF-induced Nrf2 protein expression

**Figure 4:**
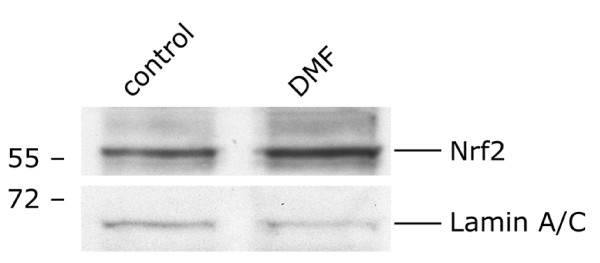
**DMF-induced expression of Nrf2 protein in microglia. **One hour after stimulation of microglia with DMF, nuclear proteins were isolated and separated using SDS-polyacrylamide gel electrophoresis, western blotted and probed with anti-Nrf2, and reprobed with anti-Lamin A/C (as a loading control) antibodies.

Encouraged by a recent publication [30] about the possible involvement of Nrf2 in the regulation of neuroinflammation, we analyzed DMF-induced Nrf2 signalling by western blotting. As shown in Fig. 4, treatment with DMF caused an increase in Nrf2 protein content in nuclear extracts of microglia (after 1 h).

## Discussion

Dimethylfumarate influences a variety of cell types and molecules participating in inflammatory conditions. Clinical studies investigating the mode of action of DMF in MS show that it depletes CD4^+ ^and CD8^+ ^T cells. This is caused by the ability of DMF to induce apoptosis in activated human T-cells [31].

Moreover, there is a shift from Th1- to Th2-cells regarding cytokine production: While the "Th1 pattern" is characterized by a proinflammatory response with increased synthesis of interleukin (IL) 2, TNF-α and IFN-γ; the cytokines IL-4, IL-5 and IL-10 are produced by Th2-cells [32].

While the inflammatory infiltrate in active MS lesions consists mostly of a perivascular accumulation of CD4^+ ^and CD8^+ ^T cells and B cells, it eventually includes microglia in the lesion center that stain positively for ingested myelin (for review see [33]). More pieces of evidence for microglial activation early in the disease course come from autopsy studies of patients suffering from relapsing-remitting MS, who died during or shortly after a relapse: Activated microglia and apoptotic oligodendrocytes are found in the absence of leukocytes [34]. Many of the studies regarding CNS adaptive immunity have focused on hematopoietically-derived microglia, which are able to take up antigens and present them to T cells.

Although they are derived from neuroectodermal lineage, astrocytes also function as antigen-presenting cells. Astrocytes express major histocompatibility complex (MHC) class I and class II molecules in vitro [35]. Functional studies indicate that IFN-γ-treated astrocytes are able to activate both CD8+ [36] and CD4+ T cells [37]. Astrocytes also have the potential to secrete inflammatory cytokines such as IL-1β, IL-6, and TNF-α [21]: Because the disease is initiated by T cells, it is likely that astrocytes are activated to release cytokines in response to T cell-derived factors and therefore, would perpetuate immune-mediated demyelination.

In this study we examined how DMF influences microglial and astroglial cells in vitro. To induce microglial and astroglial activation, LPS, a bacterial endotoxin and generally accepted inducer of pro-inflammatory properties, was added to experimental and positive control groups. In an animal model of MS local injection of LPS induces microglial activation through toll-like receptors (TLR) resulting in inflammatory demyelinating plaques [38].

A major finding of this study is that DMF negatively regulates the synthesis of NO in microglia and astrocytes in vitro as compared to LPS-activated positive controls. Expression of inducible NO synthase is abundant at the edges of MS lesions [14]. NO reacts with superoxide to from peroxynitrite, a marker for the formation of reactive nitrogen species, which nitrosylates amine and sulphate side groups on proteins and lipids. Nitrolysine is recognized as a marker of myelin damage and is found in MS plaques as well.

Activated microglia [12] and astrocytes [21] can release TNF-α, which is a potent pro-inflammatory cytokine, and blockage of microglia-dependant TNF-α delivery suppresses inflammation and demyelination in EAE [39]. It is noteworthy that in our in vitro model DMF reduced the synthesis of TNF-α, IL-1β and IL-6 in LPS-activated microglia and astrocytes as well. Ingestion of myelin debris by microglia is a pathological hallmark of active MS lesions. In adult humans, microglia that are actively phagocytising myelin vesicles secrete proinflammatory cytokines (TNF-α, IL-1β and IL-6) and undergo oxidative bursts [40]. These data are substantiated by recent findings using cytokine-stimulated airway smooth muscle cells after TNF-α-stimulation. While Seidel et al. [41] showed a significant reduction of IL-6 by DMF in a dose-dependent fashion which was due to inhibition of nuclear-factor-kappa B (NF-κB), we decided to assess a possible influence of DMF on Nrf2. The activation of Nrf2, a transcription factor possibly involved in neuroprotective responses (for recent review see [42]), supports the notion that Nrf2 is involved in the anti-inflammatory activity of DMF and, therefore, could be a promising new potential therapeutic target.

Summarizing our findings, DMF, which exerts anti-inflammatory as well as Nrf2-activating properties, might positively influence the course of relapsing-remitting MS by inhibiting microglial and astroglial activation and subsequent release of pro-inflammatory neurotoxic mediators. Oral formulations of disease-modifying drugs have advantages in convenience and will be preferred by most patients. However, we will have to await the results from ongoing large phase III trials to establish the place of DMF in the treatment of MS.

## Conclusion

We demonstrate that DMF inhibits a proinflammatory response characterised by increased microglial and astrocytic synthesis of NO, IL-1, IL-6 and TNF-α in an in vitro model of brain inflammation. The microglial mechanism involves the ERK transduction pathway and the activation of Nrf2, and may be highly relevant to the neuroprotective effect of DMF in MS.

## Competing interests

The authors declare that they have no competing interests.

## Authors' contributions

HW initiated the project and wrote the paper. JS participated in the design of the study and prepared the cell cultures. UR is a post doctoral fellow who orchestrated and carried out the work with the help of MRY. UM carried out the RT-PCR and helped to draft the manuscript. RL is the Principal Investigator and Head of the laboratory and the group, and conceptualized the experiments. All authors have read and approved the final manuscript.
